# Harnessing indigenous bacterial defenders to improve disease resistance and plant health in vegetable *Brassica* crops

**DOI:** 10.1093/sumbio/qvag025

**Published:** 2026-06-05

**Authors:** Ganesh G Madakaripura Rangaswamy, Snehalatha Pasupuleti, Jeanine Umfuyisoni, Toni A Chapman, Jonathan M Plett, Uffe N Nielsen

**Affiliations:** Hawkesbury Institute for the Environment, Western Sydney University, Hawkesbury Campus, R2, Locked Bag 1797, Penrith, NSW 2751, Australia; Hawkesbury Institute for the Environment, Western Sydney University, Hawkesbury Campus, R2, Locked Bag 1797, Penrith, NSW 2751, Australia; Division of Nematology, ICAR-Indian Agricultural Research Institute, New Delhi, India; Hawkesbury Institute for the Environment, Western Sydney University, Hawkesbury Campus, R2, Locked Bag 1797, Penrith, NSW 2751, Australia; Department of Primary Industries and Regional Development, Elizabeth Macarthur Agricultural Institute, Private Bag 4008, Narellan, NSW 2567, Australia; Hawkesbury Institute for the Environment, Western Sydney University, Hawkesbury Campus, R2, Locked Bag 1797, Penrith, NSW 2751, Australia; Hawkesbury Institute for the Environment, Western Sydney University, Hawkesbury Campus, R2, Locked Bag 1797, Penrith, NSW 2751, Australia

**Keywords:** biocontrol, microbiome, plant-diseases, rhizobacteria, sustainability

## Abstract

Vegetable crops in the Brassicaceae family have significant economic and nutritional value, supporting livelihoods worldwide. Despite ongoing efforts to control pathogens, infections continue to degrade crop quality and yield. Rising disease incidence, coupled with restrictions on chemical treatments and limited alternatives, has heightened the need for new, sustainable disease management approaches. The plant microbiome plays a crucial role in sustaining plant health and resilience under stress. Harnessing the microbiome provides an environmentally friendly means to enhance crop production by improving disease resistance. This review summarizes knowledge on how beneficial bacteria could be used to enhance plant health and disease resistance in *Brassica* crops, highlighting a preference for indigenous bacteria adapted to local growing conditions. We explore the composition and dynamics of the *Brassica* bacterial microbiome, detailing functional characteristics, biocontrol mechanisms, and potential approaches to optimize the balance between *Brassica* growth and defence under stress. We discuss methodologies for identifying and characterizing beneficial bacteria, their applications and management, and emphasize challenges hindering broad implementation. This synthesis shows that utilizing indigenous *Brassica*-associated bacteria through careful isolation, multitraits assessment, and the development of region-specific consortia offers the most reliable path to sustainable disease management. This approach links ecological adaptation with practical agricultural applications.

**Sustainability Statement**

This review advances SDG 2 Zero Hunger by emphasizing the benefits of utilizing beneficial bacteria to improve crop yields, nutrient-use efficiency, and disease resistance in *Brassica* production systems. Bacterial-based methods reduce reliance on chemical pesticides and fertilizers, supporting safer, more environmentally friendly food systems and promoting sustainable agricultural practices. These methods also align with broader sustainability objectives, including SDG 3: Good Health and Well-being, SDG 13: Climate Action, and SDG 15: Life on Land, by promoting alternatives to chemical inputs that protect both human health and the environment.

## Introduction

Vegetable crops in the Brassicaceae family, also known as cruciferous crops, are cultivated and consumed worldwide. Common *Brassica* vegetables in the household include cabbage, cauliflower, broccoli, and Brussels sprouts. All of these are varieties of *Brassica oleracea*, and possess significant health benefits, primarily due to their high content of glucosinolates, a class of phytochemicals predominantly found in *Brassica* species (Zhang et al. 1994). The distinct flavour of these vegetables is due to the presence of a chemical called 'isothiocyanate' (ITC), which has been shown to have anticancer activities and can inhibit several soil-borne pathogens (Smolinska et al. 2003, Alibrahem et al. 2025). In addition, Brassicaceae crops contain high levels of phenolics, carotenoids, vitamins, and minerals, which are essential parts of the daily human diet (Ramirez et al. 2020). The FAO estimates that the total gross production value for broccoli, cabbage, and cauliflower exceeded USD 32.3 billion in 2020 (FAO 2023). However, notable diseases in vegetable brassicas, including bacterial soft rot, black rot, clubroot, damping off, downy mildew, powdery mildew, white blister, and white mold cause substantial yield losses, leading to a decline in the production and quality of these vegetables (Greer et al. 2023).

The application of chemical pesticides and breeding for resistant cultivars have long been considered the most promising strategies for controlling plant diseases caused by microbes (Ayaz et al. 2023). While chemical pesticides are often used to control pathogens, growing awareness of potential risks to the environment and public health has led many to be deemed unsafe (Fones et al. 2020). Additionally, numerous plant pathogens have developed resistance to common chemical pesticides, complicating disease management for economically significant crops (LUCAS 2010). Using resistant cultivars is typically considered the most economical and viable option for controlling diseases. However, *Brassica* vegetables still lack effective resistant genotypes for some of the most devastating plant pathogens, such as *Xanthomonas campestris* pv. *campestris* (Xcc), the causative agent of black rot (Singh et al. 2016). Other disease management practices, such as physical treatments and in-field management, are used; however, these methods are primarily preventative and often lack specificity. To reduce the use of chemical pesticides, extracts from various plant species, including *Allium sativum, Datura metel, Parthenium hysterophorus*, and *Zingiber officinale*, have been tested and reported to reduce disease incidence due to their antibacterial properties (Jiang et al. 2025). However, the application of such extracts still requires substantial testing and may cause unintended impacts on crop performance by affecting the beneficial microbiome. To address these shortcomings, the application of beneficial bacteria has emerged as an environmentally friendly alternative. While beneficial fungal biocontrol agents (BCAs) have also been extensively investigated, this review will focus on bacteria, as they are more readily isolated and characterized and offer easier means of large-scale growth and application.

Research into the role of plant-associated microorganisms in enhancing plant health and suppressing diseases continues to expand. The practical application of this research to crop-specific contexts, such as managing pathogen-caused diseases in *Brassica* crops, is still under development, but recent advances provide valuable insights. Commercial biocontrol products often perform poorly in *Brassica* systems due to the inability of applied bacteria to overcome glucosinolate toxicity in *Brassica* tissues and due to competition from indigenous microbes for niche space in or near plant tissues. This review suggests that indigenous bacteria isolated from *Brassica* plant tissues, which are already adapted to the chemical profile of these plants, offer greater reliability. In addition, indigenous bacteria are likely to have greater abilities in colonization, nutrient mobilization, hormone regulation, and pathogen resistance within specific areas where they are isolated, allowing for region-tailored bioprotectants (Table 1). We bring together evidence from *Brassica*-related studies and carefully evaluate:

1. Functional characteristics, such as phosphorus and sulphur solubilization, ammonium and siderophore production, and plant hormone regulation, contrasting the performance of indigenous and nonindigenous strains.
2. Biocontrol strategies, including antibiosis, hyperparasitism, and quorum quenching, showing effectiveness patterns unique to pathogens affecting *Brassica*.
3. Synergistic combinations of plant growth-promoting rhizobacteria (PGPRs) and BCAs, leveraging the indigenous microbiome of *Brassica* crops.
4. Challenges in the identification and deployment of this biotechnology in managing bacterial diseases in *Brassica* crops.

**Table 1:** Evidence matrix for beneficial microbe efficacy in *Brassica* pathosystems.

Pathosystem	Crop (tissue)	Microbe/strain (origin)	Mechanism(s)	Study type	Effect size	Persistence	Reference
**Black rot** (*Xanthomonas campestris* pv. *campestris*)	Oilseed rape (leaf)	*Bacillus velezensis* X5-2 (phyllosphere isolate, indigenous)	Antibiosis (surfactin, kurstakin)	Field trial	Disease reduction: NR; Yield: NR	Phyllosphere colonization maintained	Jelušić et al. (2021)
	Oilseed rape (leaf)	*Pseudomonas orientalis* X2-1P (phyllosphere isolate, indigenous)	Antibiosis (lipopeptides)	Field trial	Disease reduction: NR; Yield: NR	Phyllosphere colonization maintained	Jelušić et al. (2021)
	Cabbage (leaf)	*Burkholderia anthina* HN-8 (crucifer field, indigenous)	Quorum quenching (DSF degradation)	Lab + greenhouse	*In vitro* inhibition: strong; Field data: NR	NR	Ye et al. (2020)
**Clubroot** (*Plasmodiophora brassicae*)	Cabbage (root)	*Pseudomonas fluorescens* DAPG+ (indigenous rhizosphere)	Antibiosis (DAPG) + ISR	Field trial	Severity ↓60%; Yield ↑~15%	High (qPCR > 60 days)	Mishra et al. (2022)
**Alternaria leaf spot** (*Alternaria brassicicola*)	Cabbage (leaf, cotyledon)	*Streptomyces* MBCN152-1 (cabbage isolate, indigenous)	Hyperparasitism (mycelial coiling, appressorium prevention)	*In vitro* + cotyledon assay	Germination inhibition: 100%; Field data: NR	NR	Shimizu et al. (2022)
	Cabbage (leaf)	*Trichoderma atroviride* IMI 206040 (commercial)	Mycoparasitism (chitinases, glucanases)	Field trial	Severity ↓40%; Yield: no change	Low (failed long-term establishment)	Turkan et al. (2023)
	Cabbage (leaf)	*Trichoderma* spp. + organic amendments (local isolates)	Mycoparasitism + competition	Field trial	Severity ↓35%; Head weight ↑12%	NR	Singh et al. (2024)
**Sclerotinia stem rot** (*Sclerotinia sclerotiorum*)	Canola (stem, leaf)	*Pseudomonas fluorescens* (rhizosphere isolate, indigenous)	ISR (JA/ET pathways)	Greenhouse trial	Severity ↓50–70%; Yield: NR	Medium (rhizosphere colonization)	Li et al. (2011)
**Fusarium wilt** (*Fusarium oxysporum*)	Chinese cabbage (root)	*Veronaeopsis simplex* (endophyte, indigenous)	Competition (space, siderophores)	Greenhouse trial	Disease incidence ↓55%; Biomass ↑20%	High (endophytic)	Khastini et al. (2012)
**General growth/stress**	Canola (whole plant)	*Bacillus subtilis* + biochar	Hormone regulation + stress tolerance	Greenhouse trial	Height ↑18%; Biomass ↑25% under drought	NR	Gul-Lalay et al. (2024)
	Chinese cabbage (whole plant)	PSM *Yangling 6* (rhizosphere isolate, indigenous)	P solubilisation	Field trial	Growth ↑significant; Soil fertility ↑	High (adapted to local pH)	Wang et al. (2017)
	Mustard (whole plant)	SOB consortium: *Burkholderia cepacia, Enterobacter cloacae* (rhizosphere isolates, indigenous)	S oxidation, N/S uptake	Field trial	Yield ↑15–20%; N/S uptake ↑	High	Chaudhary et al. (2022)

NR, Not Reported; DSF, Diffusible Signal Factors; ISR, Induced Systemic Resistance; qPCR, quantitative Polymerase Chain Reaction; ET, Ethylene; N, Nitrogen; S, Sulphur.

A review of recent studies highlights the ongoing benefits of indigenous strains for longevity and their dual advantages of growth and disease resistance over commercial options (Table 1).

## The plant microbiome: a key driver of plant health and resilience

Plants live in intimate association with many microorganisms, including bacteria, fungi, and oomycetes. These microorganisms can be beneficial, commensal, or pathogenic, and together they constitute the plant microbiome (Ali et al. 2023). Many fungi and bacteria are considered ‘beneficial’ for their ability to promote growth by supplying plants with compounds such as phytohormones; mineralizing, or solubilizing essential nutrients such as nitrogen, phosphorus, sulphur, and potassium; and facilitating nutrient uptake from the soil (Olanrewaju and Babalola 2022). The plant microbiome exhibits a degree of spatial compartmentalization (Lundberg et al. 2012, Jin et al. 2017). Microbes on plant surfaces are classified as epiphytes (Koskella 2020), while those that live within tissues are endophytes (Compant et al. 2021), and microbes found in the soil closely associated with the root (i.e., the rhizosphere) are labelled as rhizosphere microbes (Zhang et al. 2017). Plants utilize root exudates, which are primarily plant metabolites, as a major form of communication among these compartments, thereby fostering unique microbial communities in each niche (Badri and Vivanco 2009, Rizaludin et al. 2021). Epiphytes settle onto plant tissues after being transported by air, water, pollen, and soil, whereas endophytes, whose spores are transported on similar vectors, enter the plant system via natural openings or entry points, such as the stigma on flowers, spaces between cells or wounds, and spread to different plant tissues through the apoplast, cell-to-cell connections, or the vascular system (Compant et al. 2011, Sessitsch et al. 2023, De Mandal and Jeon 2023). In contrast, the microbial community in the rhizosphere is primarily derived from nearby bulk soil and is heavily influenced by root exudation patterns (Philippot et al. 2013, Sasse et al. 2018). However, the microbial community composition in the rhizosphere is not only regulated by selection pressure. Dispersal limitations can also prevent the establishment of beneficial microorganisms in the rhizosphere (Martiny et al. 2006, Bahram et al. 2018). This is one of the major reasons to apply PGPRs. In this way, the limitations on the natural recruitment of beneficial microorganisms can be overcome, thereby promoting plant growth (Lugtenberg and Kamilova 2009, Compant et al. 2010).

Plants influence the composition of beneficial microbes that help mitigate biotic or abiotic stress, both directly by synthesizing and secreting carbon-rich compounds and proteins that attract microbes to their tissues and indirectly by regulating soil structure, pH, and oxygen availability (Yang et al. 2025). In agricultural systems, the crop microbiome is influenced not only by plant traits but also by the altered soil conditions and the microorganisms associated with the seeds (Badri and Vivanco 2009, Dennis et al. 2010, Philippot et al. 2013, Plett et al. 2017, Rizaludin et al. 2021). Factors that control the extent of plant influence on the microbiome include host characteristics such as species, genotype, age, developmental stage, and health (Compant et al. 2019). Additionally, microbe–microbe interactions within their compartment are crucial for determining the structure of the microbiome, and thereby, these interactions significantly impact plant health (Stengel et al. 2022).

Given their role in promoting plant health, a whole bio-inoculants industry has emerged to identify, culture, and produce microbial inoculants on a commercial scale. To date, this industry is valued at USD 3.5–5.2 billion and is expected to grow by 7.5%–9.5% through 2033 (Verified Market Reports 2025), highlighting robust commercial confidence and ongoing demand. There have been major success stories using this approach, including for *Brassica* crops. For example, the application of liquid biofertiliser (*Chlorella fusca* CHK0059) to Chinese cabbage (*Brassica rapa* L. ssp. *pekinensis*) notably improved vegetative growth, resulting in longer, broader leaves; increased fresh and dry weights; and enhanced antioxidant activity, as evidenced by higher total phenolic and flavonoid levels (Le et al. 2025). Additionally, PGPR that produce ACC deaminase led to a 50% increase in canola seed germination under salt stress, improved the activities of hydrolytic enzymes (such as amylase, invertase, and protease), and decreased ethylene production by 35%–41% in stressed seedlings (Siddikee et al. 2015, Heydarian et al. 2016). However, there are also increasing examples of products that do not meet expectations. For instance, the effectiveness of the commercial BCA, *Bacillus subtilis*, appears to be limited in *Brassica* systems, where typical rhizosphere colonizers struggle to thrive due to the distinct biochemistry of *Brassica* species (Greer et al. 2023). These differing results emphasize that although bio-inoculants can provide significant physiological and quality improvements, their effectiveness varies widely depending on the context, and disease suppression does not necessarily lead to increased yield. Importantly, this highlights the need to more effectively match product choice, formulation, and application with crop–pathogen–environment pairings rather than presuming consistent advantages.

There are many reasons commercial products do not perform well as expected, including low viability of propagules during storage, environmental stressors such as extreme pH levels, competition with existing native microbial communities, and diminished effectiveness when non-native/introduced microbes encounter unfamiliar soil conditions (Thomsen et al. 2021, Zhou et al. 2024). Repeated failures across different agricultural systems show that successful microbial integration requires more than lab viability. It also requires maintaining ecological balance with modern microbial communities, whether in the soil, on the leaves, or inside the plant. As a result, research is shifting to determine whether indigenous microbes recruited by a plant in a specific environment or soil type have advantages over introduced microbes in disease management practices.

## Why focus on indigenous beneficial microbes?

Over evolutionary history, plants and the microorganisms that coexist with them have coevolved, forming finely tuned partnerships that bolster plant resilience and ecological balance in local environments (Lyu et al. 2021). The close nature of these associations promotes ecological stability by improving nutrient uptake and disease suppression (Kumar and Gopal 2015, Xia et al. 2022). However, crop domestication has significantly altered these historically co-evolved associations. For instance, present-day *Brassica* crops have developed from wild *Brassica species*, including *B. cretica* and *B. oleracea*, in the Eastern Mediterranean ~3000 to 3300 years ago, with *B. oleracea* giving rise to present-day *Brassica* vegetable crops, including cabbage, broccoli, cauliflower, and Brussels sprouts. These crops are now grown worldwide under conditions different from their native habitats. Due to geographic separation, therefore, these crops can no longer associate with the beneficial microbes they co-evolved with, resulting in increased dependence on external inputs such as fertilizers and pesticides for crop management compared to their wild varieties (Soldan et al. 2021).

The use of commercial bioproducts has emerged as an innovative solution for re-establishing beneficial interactions between plants and microbes in agricultural systems. However, these introduced microbes may be incompatible with the host plant and may struggle to establish and persist in cropping systems. The shortcomings of commercial bioproducts suggest a more refined strategy: utilizing the natural recruitment methods that plants have developed to attract beneficial microbes adapted to specific agricultural crops and environmental conditions. In this context, we define 'indigenous' (or local) microbes as locally occurring species that have adapted to specific agricultural fields, areas, or soil types, rather than those that co-evolved with the crop species in its place of origin. In practical terms, indigenous microbes are identified by isolating them from the local environments where the target crops grow. This process includes field sampling, followed by culturing or sequencing the microbial communities. Researchers can also use genetic or functional analyses to confirm how these microbes adapt to local soil, climate, and management practices. For instance, isolating microbes from the field and characterizing their genomes can show if they are part of the established microbial community in that specific area. These microbes are likely better suited to their local environments, and leveraging them acknowledges that crops such as domesticated *Brassica* varieties may not have co-evolved with local microorganisms. Plants have evolved advanced systems that attract microbes through root exudates and chemical signalling, which can be applied for the selective cultivation of the microbiome. Therefore, while introduced bacterial species may show comparable functional characteristics and activities, indigenous microbes are likely to engage more effectively with host plants. Recent studies support the theory that indigenous microbes associated with *Brassica* typically offer longer-lasting benefits and more reliably integrate plant growth enhancement with disease resistance compared to commercial strains (Table 2). This emphasizes the importance of using indigenous microbial resources to create sustainable, microbe-based methods for managing specific crop diseases.

**Table 2:** Comparison of effect of indigenous vs. introduced/commercial strains in *Brassica* systems.

Aspect	Indigenous/local strains	Introduced/commercial strains	Remarks	References
Field disease control	*P. fluorescens* DAPG+ (cabbage rhizosphere): clubroot severity ↓60%, yield ↑15% (field) *Streptomyces* MBCN152-1 (cabbage isolate): *Alternaria* germination 100% inhibition (cotyledon assay) *B. velezensis* X5-2/*P. orientalis* X2-1P (oilseed rape phyllosphere): Xcc suppression (field	*Trichoderma atroviride* IMI 206040 (commercial): *Alternaria* leaf spot ↓40%, no yield change (field; Turkan et al. 2023)	Indigenous microbes have a stronger and more consistent effect in *Brassica*-specific trials.	Mishra et al. (2022), Shimizu et al. (2022), Jelušić et al. (2021)
Persistence/ colonization	*Yangling 6* PSM (Chinese cabbage rhizosphere): high persistence (>60 days qPCR, adapted to local pH) *P. fluorescens* endophyte (oilseed rape): strong root/rhizosphere colonization greenhouse + field	Commercial *B. subtilis*/*Trichoderma*: low persistence, failed long-term establishment due to glucosinolates/competition Commercial BCAs: poor field colonization across crops	Indigenous microbes consistently do better colonization in *Brassica* crops	Wang et al. (2017), Lally et al. (2017), Greer et al. (2023), Turkan et al. (2023), Berendsen et al. (2012)
Yield/biomass	SOB consortium (*Burkholderia, Enterobacter*; mustard rhizosphere): yield ↑15–20% (field); *P. fluorescens* consortium (oilseed rape): biomass/height ↑ (field)	*B. subtilis* + NPK (commercial): yield ↑ but less than indigenous consortia in comparable canola trials; *Trichoderma* seed coating: no yield benefit despite disease control	Indigenous microbes offer benefits for both growth and disease suppression.	Chaudhary et al. (2022), Lally et al. 2017, Turkan et al. (2023)
Null/negative results	Some indigenous consortia: vegetative growth ↑ but no yield gain early season (field canola); Multistrain indigenous mixes: inconsistent against nontarget pathogens	*B. subtilis*/*Trichoderma* products: variable/no significant reduction vs Xcc, *Alternaria* (multiple trials)	Both have limitations. Commercial inoculants have more frequent field failures.	Lally et al. (2017), Jelušić et al. (2021), Villavicencio-Vásquez et al. (2025)

## The *Brassica* microbiome

Brassicas originated in the Eastern Mediterranean as natural biofumigants, producing glucosinolates that, when the plant tissue is damaged, break down into isothiocyanates, which are harmful to many pathogenic microorganisms. This trait makes them effective rotation crops for controlling soil-borne pathogens, nematodes, and weeds (Gantait et al. 2024). Nonetheless, this evolutionary adaptation has led to plants that discourage some beneficial microbial partnerships typically used in sustainable agriculture. For example, brassicas exhibit molecular mechanisms that hinder colonization by arbuscular mycorrhizal fungi (AMF), indicating that AMF are unlikely to function as bioinoculants for crop production or protection. Research on 55 Brassicaceae species found that nearly all retain their nonmycorrhizal status, and when AM colonization does occur, it frequently diminishes plant growth (Wang and Qiu 2006). Recent evaluations confirm this ongoing trend. They connect it to the absence of key symbiotic genes and to chemical suppression. No effective mutualism was observed throughout the family (Delaux et al. 2014, Trautwig et al. 2023). Nevertheless, recent studies suggest that cultivated brassicas foster many classes of beneficial bacteria that enhance plant health and defend against infections (Lally et al. 2017, Oulebsir-Mohandkaci et al. 2021, AbdElgawad et al. 2023, Hasan et al. 2023). Therefore, successful microbial approaches should focus on bacteria and fungi that do not form mycorrhizae, as these are better suited to *Brassica* environments. However, this review primarily concentrates on beneficial bacteria, particularly PGPRs and BCAs.

The *Brassica* microbiome consistently shows a high prevalence of specific bacterial phyla. For instance, Wassermann et al. (2017) found that the microbiome of *B. oleracea* vegetables was predominantly comprised of taxa Proteobacteria (75%), Bacteroidetes (19%), and smaller proportions of Actinobacteria, Verrucomicrobia, and Firmicutes (each at 2%), which include a great diversity of plant growth-promoting bacteria. This trend is supported by research on oilseed rape (*B. napus*), where Rybakova et al. (2017) discovered a core microbiome dominated by Proteobacteria (66%), with Firmicutes (7.9%) and Actinobacteria (1.1%). Additionally, Gkarmiri et al. (2017) reported that Proteobacteria, Bacteroidetes, and Actinobacteria were the most common bacterial groups found in the roots and rhizosphere soil of *B. napus*, further emphasizing the consistent occurrence of these main bacterial lineages within *Brassica* microbiomes.

Given that brassicas are grown worldwide, often far from their Mediterranean environment, these repeated microbial patterns likely indicate that plants can attract beneficial microbes from nearby soil ecosystems or that indigenous microbes can associate with the plant. This process offers benefits such as improved nutrient uptake and enhanced disease resistance, instead of preserving old plant–microbe relationships.

## Functional attributes and biocontrol activities of beneficial bacteria

**Figure 1:**
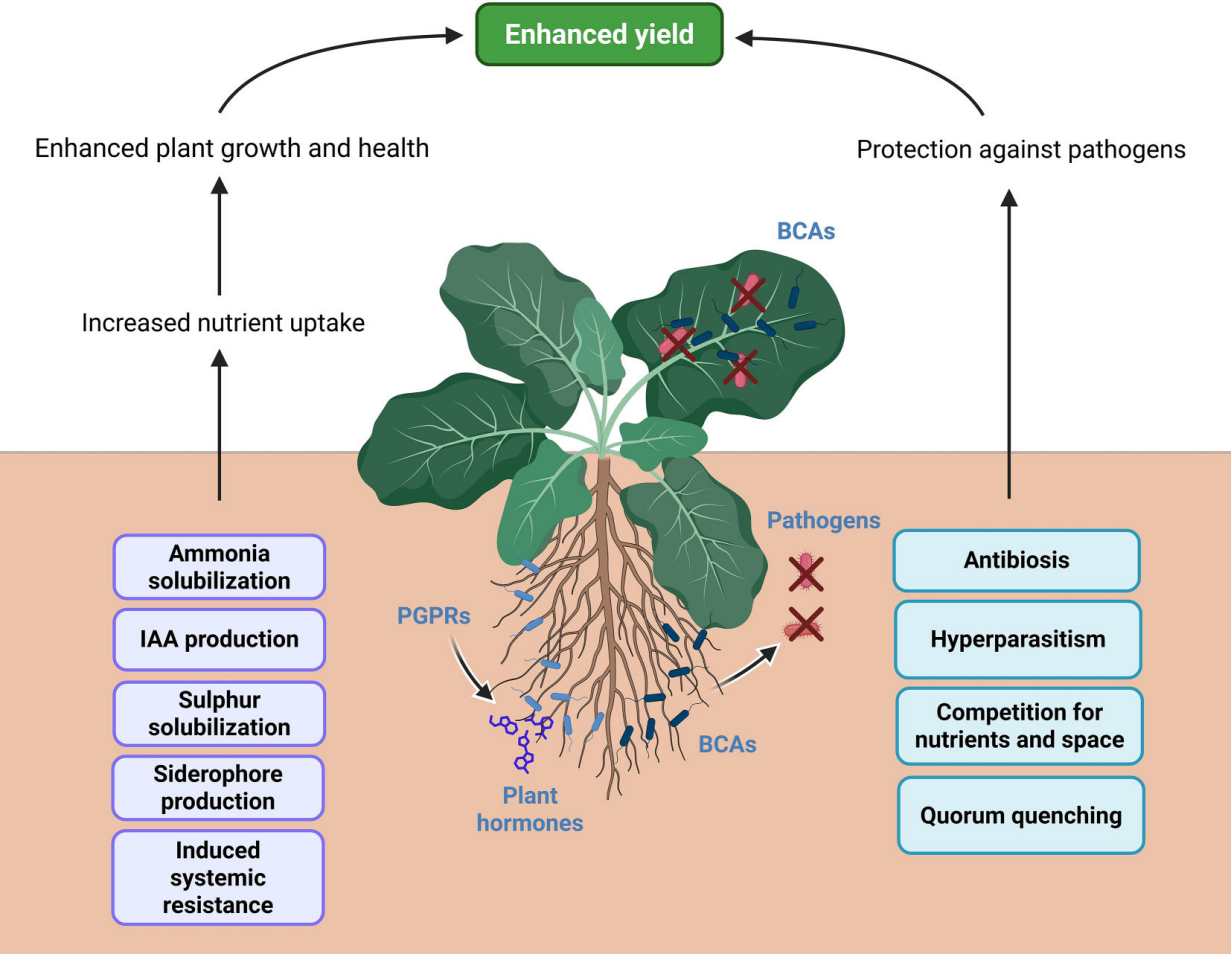
General mechanisms employed by PGPRs and BCAs. On the left-hand side are the functional attributes of PGPRs, whereas on the right-hand side are the activities employed by BCAs to combat pathogens, directly or indirectly.

Bacteria that promote plant health do so through diverse functional mechanisms and activities (Olanrewaju and Babalola 2022). Two primary modes of action are of particular interest in the context of using bacteria to suppress crop diseases: indirectly, by promoting plant immunity, and directly, by biological control. In 1989, Kloepper et al. defined PGPR as free-living soil bacteria that promote plant growth, either directly or indirectly. By enhancing plant health, these PGPRs can also help plants resist diseases by boosting their immune responses (Beneduzi et al. 2012). By contrast, BCAs directly confer disease suppression by reducing the activities and populations of plant pathogens (Pal and McSpadden Gardener 2006). For this review, we define PGPRs as beneficial bacteria that are either free-living or endophytic and enhance plant growth primarily by improving nutrient absorption and regulating plant hormones. We define BCAs as bacteria that protect plants against harmful pathogens, either directly or indirectly, irrespective of their abilities to promote growth (Pal and McSpadden 2006), meaning that a BCA can also be a PGPR (Fig. 1). In the following sections, we evaluate the key traits and mechanisms by which beneficial microbes (PGPRs and BCAs) enhance plant growth and suppress disease, with a particular emphasis on *Brassica* crops.

### Functional attributes of PGPRs that enhance plant growth in *Brassica* crops

With a focus on *Brassica* crops, this section highlights the key features of beneficial microbes. These include phosphorus and sulphur solubilization, ammonia and siderophore production, and plant hormone production. These factors ultimately improve plant nutrition, enabling a greater allocation of resources to improve plants’ defense systems. These features tackle major nutritional issues that limit *Brassica* crop productivity and disease resistance. Although there are still some direct comparisons, native microbes consistently outperform commercial or introduced strains across crops due to their better local adaptation, higher persistence, and better compatibility with native communities (Olanrewaju et al. 2017). For *Brassica*, a commercial strain, *B. subtilis* often struggles to establish within plant tissues or in proximity due to glucosinolate toxicity and competition. In contrast, rhizosphere isolates such as *Bacillus cereus* YL6, a phosphate-solubilizing bacterium, thrive and confer benefits in the field (Wang et al. 2017, Greer et al. 2023). These trends occur because indigenous strains are already well-suited to *Brassica* biochemistry. While the ecological reasoning and supporting evidence clearly show that we should prioritize locally sourced microbes for sustainable use, multi-trial data are still needed to test whether they outperform commercial alternatives.

#### Ammonium production

Nitrogen (N) is a crucial nutrient for chlorophyll synthesis and photosynthesis, both of which support plant growth and crop yield. However, most of the soil nitrogen is in organic forms that plants cannot use (Zayed et al. 2023). While inorganic fertilizers can help alleviate N limitation, they are costly, and much of the added N is often lost through leaching. Many beneficial bacteria commonly found in the *Brassica* microbiome, including members of the genera *Arthrobacter, Azospirillum, Azotobacter, Enterobacter*, and *Pseudomonas*, improve nitrogen uptake by transforming atmospheric N into usable forms for plants (Bhattacharyya and Jha 2012). Microbial mineralisation of N not only enhances plant development but also strengthens defense mechanisms; supplying NO_3_^−^ can notably increase the total phenol and flavonoid levels in leafy *Brassica*, thereby improving the plant’s defense systems (Fallovo et al. 2011). Additionally, the assimilated N can drive amino acid metabolism, further supporting defense against plant diseases, as amino acids are building blocks for many defense-related compounds, such as phytoalexins and glucosinolates (Zeier 2013). In *Brassica*, this is illustrated by glucosinolates, which are key phytoalexins produced from amino acids such as alanine, phenylalanine, and tryptophan, and their levels increase with elevated nitrogen availability (Sønderby et al. 2010). This underscores the need to identify and utilize specific beneficial bacteria from the *Brassica* microbiome that can transform inaccessible N sources into forms such as ammonium and nitrate, thereby fostering both physical and biochemical defense barriers in *Brassica* crops.

#### Phosphorous solubilization

Phosphorus (P) is the second most crucial macronutrient for plant growth and development; however, much of the soil P is in forms that plants cannot access (Cade-Menun et al. 2010, Bhattacharyya and Jha 2012). Similarly, most phosphate fertilizers applied to agricultural fields are rapidly converted into insoluble complexes in soil (Chien et al. 2010). Phosphate-solubilizing microorganisms (PSMs) offer a viable solution for recovering these inaccessible stocks by converting insoluble soil P into plant-available forms through acidification (Khan et al. 2009, Richardson et al. 2009). Research comparing various P sources indicates that the effectiveness of P-solubilizing bacteria is influenced by both the specific bacterial strain and soil properties. The same strains exhibited different effects on P fractions in acidic soils compared to alkaline soils, suggesting that PSM communities adapted to local conditions may be more effective than commercial inoculants (Long and Wasaki 2023). Consequently, utilizing native PSMs from *Brassica* rhizospheres offers a promising strategy to alleviate P limitations in these production systems.

#### Sulphur solubilisation

Sulphur (S) is another essential macronutrient for plants and is particularly important for *Brassica* glucosinolate biosynthesis (De Pascale et al. 2007). While plant leaves can absorb gaseous SO_2_ and H_2_S, the primary source of S accessible for plants comes from sulphate (SO_4_^2−^) present in the soil (Telman and Dietz 2019). S-oxidizing bacteria (SOB) from the bacterial genera *Acidithiobacillus, Thiobacillus, Enterobacter, Klebsiella, Phytobacter*, and *Pseudomonas* can convert S into a form of sulphate that plants can utilize (Gijs Kuenen 1975, Shinde et al. 2022). A study by Chaudhary et al. (2022) examined SOBs, including *Burkholderia cepacia, Enterobacter cloacae*, and *Klebsiella oxytoca*, isolated from mustard fields and found that they significantly enhanced N and S uptake in mustard plants in field experiments. A recent study identified SOBs, including *Enterobacter ludwigii, Enterobacter hormaechei*, and *Bacillus sp*., from the mustard rhizosphere, demonstrating that these strains can oxidize sulphur in both laboratory and field settings. The research also shows that the application of these bacteria improved crop yields and promoted soil health by reducing reliance on chemical fertilizers (Vishnu et al. 2024). These locally adapted SOBs offer specific advantages for *Brassica* cultivation by improving S availability. This availability is essential for the synthesis of defense compounds while promoting plant growth.

#### Siderophore production

Iron (Fe) bioavailability is limited in aerobic soils, despite its abundance. This limitation affects respiration, photosynthesis, and nitrogen and sulphur uptake in *Brassica* species (Ma 2005, Touraine et al. 2019). Many bacteria synthesize low-molecular-weight siderophores with high affinities for ferric ion (Fe³⁺), helping dissolve Fe so plants can absorb it (Neilands 1981, Hider and Kong 2010). For example, 24 bacterial isolates from the rhizosphere of mustard (*Brassica juncea*) exhibited significant siderophore production indices (SPIs of 1.03–1.70), suggesting their potential to improve plant health under iron-deficient conditions (Abirammi et al. 2018). Nevertheless, studies of siderophore-producing bacteria in *Brassica* systems remain limited, highlighting a significant knowledge gap that requires further research to fully understand and utilize these microbes.

#### Plant hormone regulation and induction of systemic resistance

The regulation of plant hormones influences all aspects of plant growth and development. Importantly, some PGPRs can generate or influence these hormones, which yield multifaceted benefits: promoting vegetative growth through auxins and gibberellins (GA), boosting resilience to environmental stress via jasmonic acid (JA), and activating immune responses through ethylene regulation (Das et al. 2025). By carefully modulating hormonal levels, PGPRs can tailor plant growth and development, even under stress.

Indole-3-acetic acid (IAA) is a ubiquitous natural auxin found in plants that regulates and coordinates numerous aspects of growth and development (Zhao 2012, Fu et al. 2015). Rhizosphere bacteria transform root-exuded tryptophan into IAA. This process increases root branching and oil content in canola (*B. napus*; (Asghar et al. 2004) and induces specific changes in gene expression, including upregulation of auxin response factors and downregulation of stress-response genes (Liu et al. 2024).

Ethylene is a crucial plant hormone that plays a key role in growth and development; however, excessive ethylene production under stressful conditions, such as salinity, drought, and pathogen stress, triggers senescence (Saleem et al. 2007, Bhattacharyya and Jha 2012). ACC deaminase-producing PGPRs, such as *Pseudomonas, Bacillus*, and *Azospirillum*, break down the ACC precursor, thereby reducing the ethylene levels (Nadeem et al. 2007). In canola (*B. napus*), *Pseudomonas putida* UW4 reduced stress-induced ethylene production, increasing root length and biomass while downregulating ethylene-responsive genes (Walton et al. 2012). GAs, derived from the terpenoid biosynthetic pathway, are essential for regulating processes such as cell division, elongation, and seed germination (Bhat et al. 2023). GA-producing PGPR strains, *Priestia aryabhattai* KW05 and *Pseudomonas frederiksbergensis* KW07, have been found to significantly improve the growth of broccoli plants under salt stress by increasing root elongation and resource uptake, helping plants thrive in adverse conditions. (Woo et al. 2023).

In addition, PGPRs such as *Pseudomonas fluorescens* strains isolated from the B. napus rhizosphere have been shown to protect canola plants against Sclerotinia sclerotiorum by inducing ISR and activating JA/ET-mediated defence pathways (Li et al. 2011). Consequently, utilizing ISR-inducing microbes in *Brassica* crops not only yields effective disease control but also provides a sustainable approach to enhancing resilience while maintaining crop productivity under various stress conditions. Thus, hormone regulation by indigenous PGPRs can help plants thrive under stress and resist diseases by promoting root growth through auxins and GA, reducing stress-induced senescence with ACC deaminase, and activating JA/ET-mediated ISR against pathogens. Although research on *Brassica*-specific hormone regulation is limited, these mechanisms highlight the importance of microbe-host interactions. Indigenous *Brassica* PGPRs are likely to use hormone-ISR effects more effectively than commercial strains. This suggests that focused experiments are necessary.

### Biocontrol activities of beneficial microbes

Many of the beneficial bacteria in the plant microbiome exhibit biocontrol properties (Etesami 2025), representing a vast underutilized resource, particularly for indigenous *Brassica* microbes that thrive in glucosinolate-rich environments. Their capability to inhibit plant pathogens through a range of antagonistic strategies makes them valuable biological alternatives to chemical pesticides. The following examines the biocontrol functions of beneficial microbes, emphasizing their mechanisms and potential for managing diseases affecting *Brassica* crops.

#### Antibiosis, hyper-parasitism, and quorum-quenching

BCAs can inhibit the growth and proliferation of other microorganisms by producing diffusible antimicrobial compounds (Thomashow et al. 2008). Some of the most promising among them include cyclic lipopeptides such as surfactin, iturin, and fengycin derived from *Bacillus* species, which target and disrupt the cell membranes and biofilms of pathogens (Ongena and Jacques 2008, Caulier et al. 2019). Additionally, 2,4-diacetylphloroglucinol (DAPG) producing *Pseudomonas fluorescens* strains significantly reduced the *Plasmodiophora brassicae* infection through antibiosis and ISR in cabbage (*B. oleracea*) plants (Mishra et al. 2022), while oilseed rape phyllosphere isolates (*Bacillus velezensis* X5-2, *Bacillus megaterium* X6-3, and *Pseudomonas orientalis* X2-1P) suppressed Xcc infection through a range of antimicrobial metabolites (Jelušić et al. 2021).

Hyper-parasitism involves BCAs enzymatically degrading pathogen cell walls to obtain nutrients (Muthu Narayanan et al. 2022). A predatory bacterium, *Bdellovibrio bacteriovorus*, parasitises many Gram-negative bacteria, including *Agrobacterium tumefaciens, Xanthomonas vesicatoria*, and *X. campestris* pv*. campestris, Erwinia carotovora* pv. *carotovora*, and *Pseudomonas syringae* pv. *glycinea* (McNeely et al. 2017). *Streptomyces* sp. strain MBCN152- 1, isolated from cabbage plants, exhibited targeted hyper-parasitism against *Alternaria brassicicola*, effectively preventing the formation of an appressorium (Shimizu et al. 2022).

Another strategy employed by some BCAs is to disrupt quorum sensing, by which many pathogens regulate virulence factors at high population densities via cell-to-cell communication (Zhang and Dong 2004, Marian and Shimizu 2019). This QS occurs with the help of diffusible signal factors in many Gram-negative bacteria, including Xcc and N-acyl homoserine lactone in *Pseudomonas* spp. (Wang et al. 2004). BCAs break down these signals via enzymes such as lactonases and chitinases, thereby preventing infection, a process termed quorum quenching (Uroz et al. 2009, Bonaterra et al. 2022). *Burkholderia anthina* strain HN-8, isolated from cruciferous crop fields, was found to degrade Xcc DSF signals, thereby inhibiting the pathogen’s virulence (Ye et al. 2020). Only a few studies have found quorum-quenching BCAs from *Brassica* microbiomes. These results set the stage for exploring the *Brassica* microbiome and identifying other beneficial bacteria with quorum-quenching abilities that target pathogens affecting *Brassica* crops.

#### Competition for nutrients and space

As another facet of their role in promoting plant health, beneficial bacteria suppress pathogens by competing for key resources (iron, carbon, and amino acids) and colonization sites (Köhl et al. 2019, McLaren and Callahan 2020). As we discussed above, how siderophore-producing bacteria can sequester Fe³⁺ for plant use, they can also capture Fe³⁺ away from other microbes, thereby depriving pathogens of this resource (Whipps 2001, Lugtenberg and Kamilova 2009). For instance, *Pseudomonas chlororaphis* YL- 1 inhibited *Xanthomonas oryzae* pv. *oryzae* under low-iron conditions (Liu et al. 2021). A comprehensive understanding of pathogen epidemiology is crucial for using this approach to disease management, as it pinpoints the stages at which nutrient and space limitations affect pathogen growth.

In the following sections, we will see how different mechanisms of these beneficial bacteria can be used to balance the growth-defense trade-off in plants, especially under stress conditions.

## Synergetic effects of combining PGPRs and BCAs: an attempt to balance plant growth-defense trade-offs

**Figure 2:**
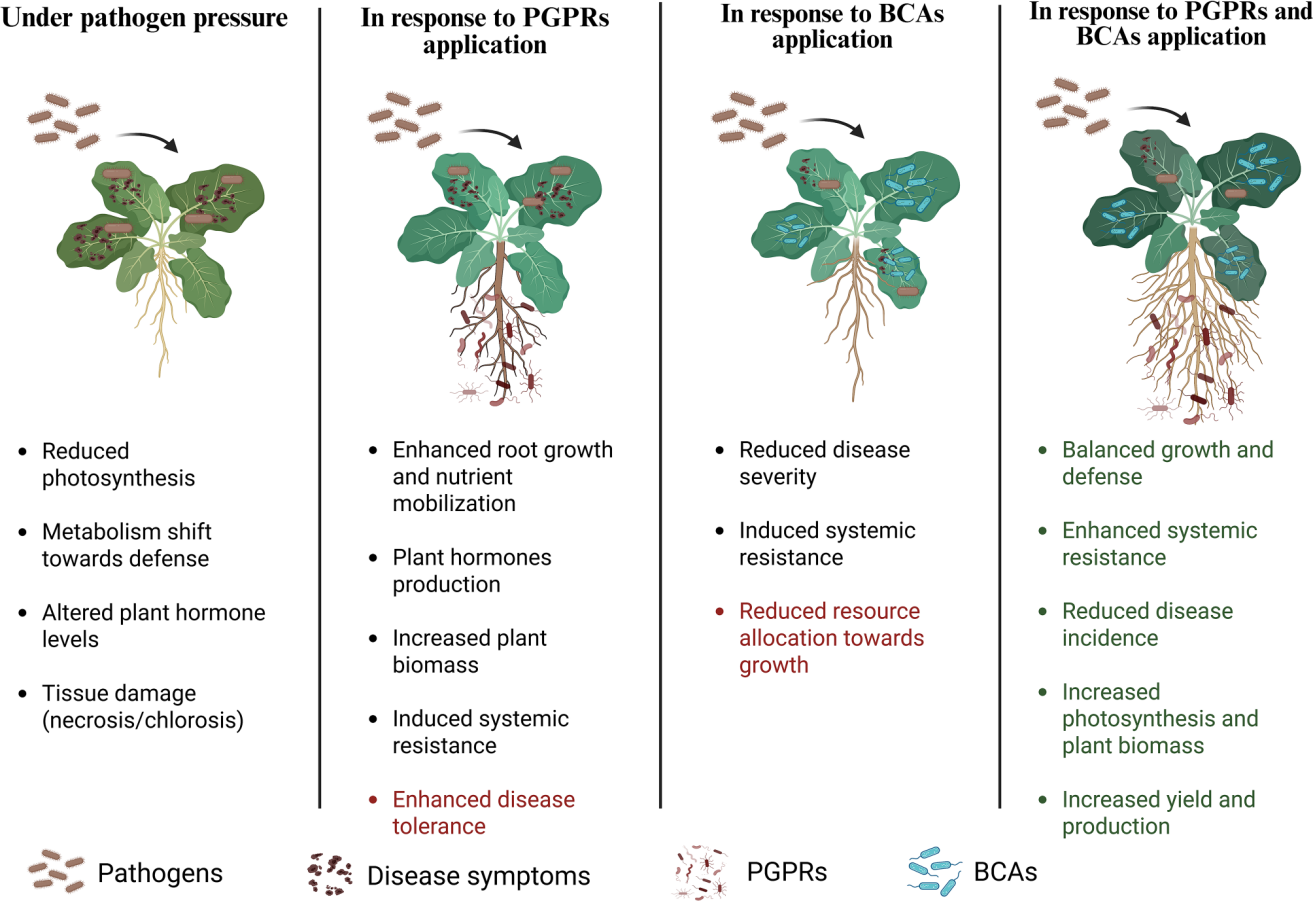
A brief overview of the beneficial effects of plant-microbe interactions during pathogen infections.

Plants deploy defensive mechanisms to cope with biotic stress, but this generally comes at the cost of growth (Lamalakshmi Devi et al. 2017). This diversion of plant resources occurs at all levels, including transcription, translation, and protein synthesis, as well as in the allocation of carbon and nitrogen for the production of defense compounds (Borges et al. 2013). In milkweed, the trade-offs between defense and growth are more pronounced under low nutrient conditions, whereas in *Arabidopsis* mutants, that maintain constant defense activation, the addition of sucrose promotes growth, suggesting that increased resource availability can facilitate both growth and defense (He et al. 2022). Therefore, balancing resource allocation between growth and defense in response to biotic stress would, in theory, optimize plant fitness and disease suppression. For instance, the activation of defense responses, where plant hormones such as SA, auxin, and glucosinolates are involved, relies largely on sulphur availability (Király et al. 2012). Thus, sufficient sulphur nutrition is essential for maintaining the production of these compounds, and supplementing this nutrient with PGPRs would be advantageous. In this context, mixtures of beneficial microbes are increasingly considered for their roles not only in improving plant yield but also in enhancing plant defense mechanisms simultaneously. As we discussed in the previous section, PGPRs enhance plant health and BCAs directly or indirectly combat pathogens, their combined use (PGPRs as soil treatments and BCAs as foliar sprays) offers a potential approach to improve the management of foliar diseases, although further studies are needed to confirm its effectiveness (Fig. 2). As BCAs, PGPRs in rhizosphere soil can also initiate low-cost ISR via jasmonate/ethylene signalling, enabling the plant to remain in a ‘primed’ state that responds rapidly to pathogen threats. This priming can boost disease resistance while minimizing metabolic costs, enabling plants to sustain or even enhance biomass growth and overall development even in stress conditions. On the other hand, targeted foliar-applied BCAs can directly compete with incoming pathogens through various mechanisms, thereby shielding the plant from infection. Because of the complementary characteristics of PGPRs and BCAs, there is growing interest in controlling foliar diseases through synergistic mechanisms involving beneficial microbes without compromising growth. We consider this approach a promising disease management strategy to reduce or avoid reliance on chemical pesticides.

One practical way to evaluate the growth-defense trade-off balance under the combined use of PGPRs and BCAs is to incorporate physiological, biochemical, and molecular parameters. Key growth parameters, such as plant height, root and shoot biomass, leaf area, and root parameters, should be measured alongside physiological parameters, including net photosynthetic rate, stomatal conductance, transpiration rate, and electron transport rate. These measurements will indicate how plants respond to the external stress under the combined treatment of PGPRs and BCAs, as these parameters are directly associated with plant productivity (Medrano et al. 2002). On the other hand, analysing disease severity, pathogen load, and expression levels of defense-related genes associated with ISR and systemic acquired resistance will help quantify defense responses in plants (Pieterse et al. 2014). Given the importance of nutrient availability to plants, it is crucial to monitor key elements that support both growth and defence. By evaluating these parameters across different microbial treatments, we can identify the best combinations of microbes that reduce disease without compromising plant growth. Additionally, using multivariate statistical methods or composite indices to combine growth and defense measurements can provide a way to describe this balance. This approach helps in choosing microbial groups that improve both plant yield and resistance to biotic stress (Walters and Heil 2007, Huot et al. 2014).

The subsequent sections will focus on techniques for identifying and characterizing beneficial bacterial communities within the plant microbiome, laying a foundation for their practical application in sustainable agricultural practices.

## Approaches for the identification and characterization of beneficial bacteria

The *Brassica* microbiome exhibits considerable diversity, with around 32 bacterial phyla found in the rhizosphere, depending on the cultivar (Taye et al. 2020). This greater diversity in the rhizosphere than in bulk soil indicates significant root-mediated selection (Laiq et al. 2024). Although this diversity offers a significant resource for discovering PGPRs and BCAs, it also presents a challenge in determining which indigenous bacterial strains are most effective in promoting plant health and which should be targeted for either direct encouragement *in situ* through management practices or for culture and use as bio-inoculants. This complexity necessitates rigorous research to explore the *Brassica* microbiome, enabling the accurate identification and characterization of beneficial bacterial groups that directly enhance plant growth and combat diseases. To support and direct research, the following section explores the primary methods, both culture-dependent and culture-independent (Sathyanarayana Reddy et al. 2016), which can be used to identify and evaluate beneficial bacteria within the *Brassica* microbiome, laying the groundwork for the identification, management, and effective use of PGPRs and BCAs either *in situ* or as inoculants.

### Culture-dependent methods

Culture-dependent methods are used to isolate potential PGPR and BCA strains from plant or soil samples by diluting and cultivating them on nutrient media to promote the growth of viable bacteria. These isolates are then characterized by assessments of colony and cell morphology, biochemical tests, and validated using molecular techniques such as 16S rDNA sequencing and phylogenetic analysis (Hayat et al. 2013, Begum et al. 2017, Vishnu et al. 2024). Although this approach recovers only a small portion (0.1%–1%) of overall microbial diversity, it typically favours fast-growing species and overlooks unculturable taxa. These methods are, however, cost-effective and necessary steps for further development of inoculants for sustainable disease management (Davis et al. 2005, Zhao et al. 2023). Recent developments are beginning to address the issue of the growth of previously unculturable taxa, such as the soil substrate membrane system designed by Ferrari et al. (2008), which has broadened the range of microorganisms that can be cultured. Despite the drawbacks of this approach, many case studies outlined above show that culturable bacteria serve as a useful initial step in formulating microbial products for agricultural use, functioning as an essential filter for identifying candidate PGPR/BCA.

### Culture-independent methods

Culture-independent methods have revolutionized soil microbial ecology, overcoming the limitations associated with culture-dependent techniques by providing taxonomic, functional, and activity-based insights into unculturable bacterial communities (Su et al. 2012, Rincon-Florez et al. 2013). In the realm of PGPR and BCAs, such information is essential for three primary reasons:

- To pinpoint beneficial functional characteristics (such as nutrient solubilization, phytohormone production, and induction of ISR) that may be lacking in the existing microbiome and could be enhanced through targeted inoculation.
- To identify possible microbial limitations, such as pathogens or antagonistic species, that could compromise the effectiveness of PGPRs/BCAs.
- To grasp the broader microbiome framework that promotes plant health, enabling the design or management of inoculant applications that integrate seamlessly with the current microbial community. By combining molecular characterisation with the selective culturing of key taxa, scientists can translate metagenomic and functional data into customized PGPR/BCA consortia or management strategies that deliver improved consistency and performance in the field.

Whole-genome sequencing enables the identification of genes associated with beneficial traits, guiding the targeted selection of the best isolates for inoculant development rather than relying solely on phenotypic screening (Almirón et al. 2025). On the other hand, amplicon sequencing of the 16S rDNA gene remains the cornerstone for targeted profiling of bacteria directly from environmental DNA (Zhao et al. 2023). Additionally, complementary multiomics approaches (including metagenomics, metatranscriptomics, metaproteomics, and metabolomics), alongside imaging and hybridisation techniques (such as Fluorescence In-Situ Hybridization and microarrays), facilitate the functional and spatial analysis of bacterial populations (Wagner and Haider 2012, Bastida et al. 2014, Castañeda and Barbosa 2017). While culture-based techniques bridge the gap between phenotype and function, utilizing culture-independent methods, such as molecular and omics data, provides a holistic understanding of microbiomes associated with soil and plants.

Overall, each method for the identification and characterization of beneficial bacteria has distinct merits and demerits; however, relying on a single method is generally insufficient (Table 3). Integrating both methods is important when selecting indigenous PGPR or BCA candidates rather than relying on standard commercial strains. Ongoing advancements and achievements in molecular techniques, bioinformatics tools, and imaging indicate the potential of our capabilities to study beneficial bacterial communities that directly affect agricultural productivity and environmental sustainability.

**Table 3:** Key features of culture-dependent and culture-independent approaches for identifying beneficial *Brassica*-associated microbes.

Approach	Main advantages	Main limitations	Typical applications in *Brassica* PGPR/BCA work	References
Culture-dependent methods	Direct access to living isolates for functional assays, genome sequencing, and inoculant production. Enables precise testing of traits such as nutrient solubilisation, hormone production, and antagonism against pathogens	Capture only a small, fast-growing fraction of total diversity. Under-represent slow-growing or host-specialised indigenous taxa. Isolation conditions can select poorly adapted strains that later perform inconsistently in the field	Initial screening of candidate indigenous PGPRs/BCAs from *Brassica* rhizosphere or root tissues. Construction and optimization of inoculant formulations. Validation of functions inferred from omics data.	Ferrari et al. (2005, 2008)
Culture-independent methods	Reveal total community structure, including unculturable and rare taxa. Link taxa with functions and plant compartments Identify indigenous core/hub taxa associated with health, stress tolerance, and disease suppression	Provides correlative rather than causal evidence. Require substantial bioinformatics capacity and careful experimental design. Do not directly yield strains for inoculation without subsequent cultivation	Profiling *Brassica* microbiomes across cultivars, sites, and managements, identifying functional gaps and keystone taxa. Guiding targeted isolation of indigenous strains for synthetic communities and tailored inoculants.	Taye et al. (2020), Bell et al. (2022), Perez-Garcia et al. (2016)

## Microbial interventions and management practices

Beneficial microbe-based interventions for disease management are considered sustainable and eco-friendly approaches. By utilizing these microbes, it is possible to control infections without compromising plant growth and development. Depending on the target pathogen and crop, they can be applied as seed treatments, soil drenches, or foliar applications. When combined with suitable agronomic practices, these microbe-based interventions will enhance crop production while successfully managing diseases. Initially, research on utilizing beneficial microbes focused on single-strain applications; however, due to the variability of field conditions, resistance to these beneficial strains developed in target pathogens, and limited competitiveness against already existing microbes, studies related to developing consortia of different beneficial microbes came into light (Trivedi et al. 2017, 2020, Mitter et al. 2019). As discussed earlier, various beneficial microbial strains may utilize distinct mechanisms of action/functions to protect plants from infections. Hence, it has been suggested that employing a combination of bacteria (consortia) can improve the effectiveness of actions by increasing their stability, expanding their range of applications across various plants, soil types, and pathogens, as well as enhancing their overall impact (Bradáčová et al. 2019). Generally, microorganisms in nature exist in complex interactions with other species and other kingdoms (Raaijmakers et al. 2009). These interactions will benefit the preparation of consortia comprising distinct species/genera, thereby enhancing their performance.

Testing the compatibility of microorganisms for use in consortia is critical, as some mechanisms of action of beneficial microbes, for instance, BCAs that produce antibiotics, may kill or reduce the activity of other BCAs. Several techniques are available for evaluating strain compatibility, each with its own set of advantages and disadvantages. Nevertheless, the most prevalent method for assessing the biocompatibility of strains and their effectiveness against specific pathogens hinges on direct antagonism observed in artificial media (De Cal et al. 2020). Given that microbial secondary metabolism is significantly influenced by nutrient availability (Sánchez et al. 2010), it has been proposed that candidates for biological plant protection should be chosen based on their *in vivo* performance rather than *in vitro* results (Köhl et al. 2011). Although the success of consortia over individual isolates ultimately relies on meticulous strain selection, compatibility assessments, and performance verification under realistic *in vivo* conditions, their formulation generally involves aggregating microbes with complementary functional characteristics (such as nutrient solubilization, hormone modulation, and pathogen suppression). We believe that microbial consortia offer a valuable approach compared to the individual application of microbes, improving the reliability, effectiveness, and ecological significance of biological interventions in sustainable agriculture.

## Challenges and future directions

Microbial interventions have become a viable approach in agriculture, offering alternatives to traditional agrochemicals by alleviating soil limitations, such as nutrient shortages, and, more recently, by reducing pathogen pressures. Although inoculants often target specific issues, such as nutrient deficiencies or diseases, recent progress highlights the need to shift towards microbial communities capable of handling multiple stressors simultaneously. In *Brassica* systems, several challenges complicate this matching process. These include the ecological complexities of glucosinolate-rich exudates and the vulnerability of inoculants under intensive management. Additionally, there are formulation limitations and issues with the economic and regulatory environment for microbial products.

**Figure 3:**
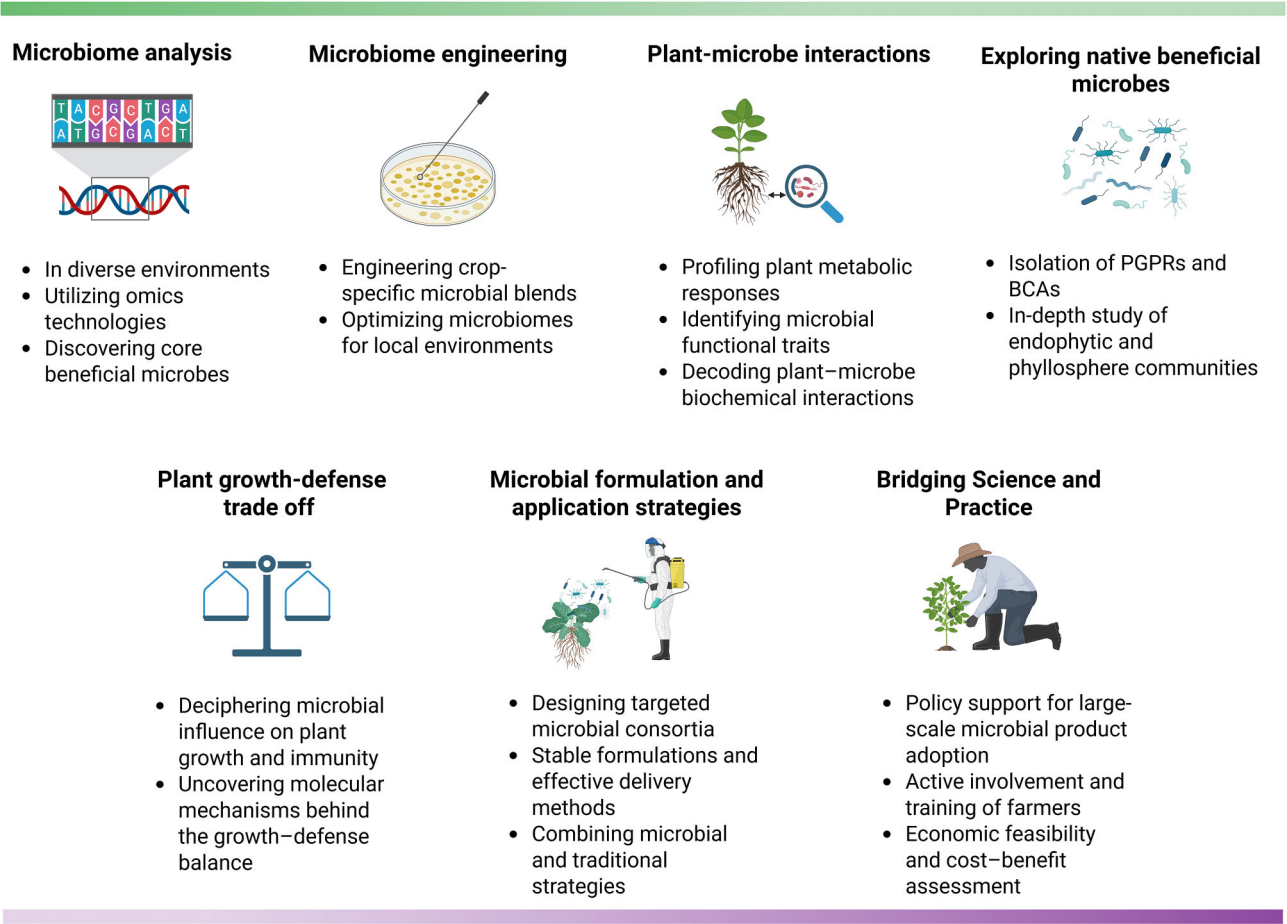
Schematic overview of potential strategies to harness beneficial microbes to enhance plant health and disease management in sustainable cropping systems.

A significant ecological obstacle is the chemically challenging environment of *Brassica* roots and shoots. High levels of glucosinolates and their breakdown products in the *Brassica* root exudates could reduce microbial colonization and survival, thereby affecting PGPR colonization and even their interoperability with BCAs (Smith and Kirkegaard 2002, Hiruma 2019). Future research (Fig. 3) should focus on utilizing modern omics technologies to conduct detailed microbiome analyses across *Brassica* production environments. Specifically, genomics could identify microbial pathways that detoxify or tolerate *Brassica*-specific metabolites such as glucosinolates. This would enable the selection of stable core beneficials for developing inoculants. Increasing attention to the variability of the plant metabolome across different management practices could reveal how factors such as fertilizer use and irrigation affect both the chemistry of *Brassica* species and their microbiome.

Intensive farming methods pose a secondary obstacle. *Brassica* crops are often grown in high-intensity management systems that rely heavily on pesticides and fertilizers, practices that can inhibit microbial communities or directly harm inoculants (Suman et al. 2022). Instead of simply pushing for “reduced inputs,” future strategies should draw on ecological theory to manage inputs such as fertilizers, pesticides, and organic amendments. This will help establish specific beneficial microbial groups. This approach creates microbiome-friendly systems from the outset, reducing dependence on agrochemicals while enabling effective inoculant performance in intensive *Brassica* production.

Developing a stable formulation is a fourth barrier that is often overlooked. The survival and effectiveness of inoculants depend on their formulation. A stable formulation ensures they reach the intended areas and protects the microbes during storage and application (Bashan et al. 2014). For *Brassica* systems, this includes developing seed coatings, soil and foliar products, and carriers that withstand common stressors such as heat, ultraviolet light, and agrochemical exposure. New methods include encapsulation techniques that release cells in response to root exudate signals, and co-formulations that use organic amendments or biochar to create suitable microsites for colonization.

Ultimately, economic and regulatory factors may limit the practical use of promising *Brassica*-specific inoculants. Bringing effective microbial products to market involves multiple stages and presents various challenges. Before obtaining a license for commercial use, they must undergo risk assessments, much like synthetic pesticides (Wilson and Jackson 2013). In the EU, products are subject to the Plant Protection Products Regulation and must receive approval from both European authorities and member states (Sundh and Eilenberg 2021). In the US, the Environmental Protection Agency (EPA) manages microbial pesticides and requires data on toxicity, pathogenicity, and effects on nontarget organisms (USEPA 1996)). In Australia, the Australian Pesticides and Veterinary Medicines Authority (APVMA) evaluates microbial products using a system similar to that for chemical pesticides (APVMA 2024). The process can be lengthy and resource-intensive, and coordination between regions is limited; approval in one area does not guarantee approval in another. Researchers and developers should expect to provide thorough documentation and plan for these requirements early on in product development.

A key goal for the future is to develop effective ways to balance plant growth and defense when using both PGPRs and BCAs. The trade-offs between growth and defense have already been well documented (Walters and Heil 2007, Huot et al. 2014). However, the application of this knowledge to microbial solutions for combating disease remains limited. Future research should combine measurements of growth, physiological responses, and defense mechanisms, to identify treatment combinations that boost disease resistance while maintaining plant productivity. Using composite indices or modelling techniques to assess this balance, and improving microbiome engineering, could help create tailored PGPR and BCA groups that enhance both crop production, productivity, and resilience under stress.

Future research should also focus on detailed cost-benefit analyses. These analyses should consider not only yield improvements but also reductions in pesticide use, improved soil health, and resilience to climate change. Support through farmer participation in regulation is vital to the successful commercialisation and incorporation of beneficial microbes into sustainable *Brassica* production systems. Finally, aligning microbial use with climate resilience and sustainability goals will embed these biological tools into future environmentally sustainable agricultural practices.

## Conclusion

This review shows that managing diseases in *Brassica* crops is more effective with indigenous microbes than with introduced ones. Because these crops release large amounts of glucosinolates and are grown under intensive farming conditions, only certain microbes survive well in these conditions. Most commercial strains fail to colonize, perform inconsistently, and have limited durability. However, naturally occurring PGPRs and BCAs, already shaped by such environments, tend to deliver steady results through multiple actions at once, unlocking nutrients, adjusting plant hormone levels, and fighting pathogens. To achieve future advancements, we need to optimize plant growth and defence through the coordinated application of PGPRs and BCAs, and to combine culture-dependent and culture-independent methods to identify key beneficial microorganisms in the *Brassica* microbiome. Linking metabolome and microbiome data to functional traits enables the creation of synthetic communities tailored to different *Brassica*-soil-climate settings. In this way, indigenous microbes associated with *Brassica* are not just an alternative to chemical controls; they are an essential part of sustainable, climate-resilient farming.

## Data Availability

No new data were generated or analysed in support of this research.
